# Withstanding psychological distress among internally displaced Yazidis in Iraq: 6 years after attack by the Islamic State of Iraq and the Levant

**DOI:** 10.1186/s40359-022-00973-8

**Published:** 2022-11-11

**Authors:** Omar S. Rasheed, Lucía López-Rodríguez, Marisol Navas

**Affiliations:** 1 University of Almería, Carretera Sacramento, S/N, La Cañada de San Urbano, 04120 Almería, Spain; 2 International Committee of the Red Cross (ICRC), Geneva, Switzerland; 3 Center of Study of Migration and Intercultural Relations (CEMyRI), Almería, Spain; 4 Artis International, Scottsdale, USA

**Keywords:** Psychological distress, Resilience, Yazidis, Conflict, Mental health

## Abstract

**Background:**

Insurgents of the Islamic State of Iraq and the Levant created a crisis that has had immediate and long-term consequences for the population in Iraq. Yazidis are among the most affected ethnos religious groups in the region. The current study focuses on investigating the level of psychological distress and its association with subjective resilience among the Yazidi minority 6 years after the attack by the Islamic State of Iraq and the Levant.

**Methods:**

The present study recruited four hundred and twenty-two Yazidi individuals (50.8% female) residing in two camps in the Iraqi Kurdistan region. In face-to-face interviews, each participant replied to different scales to measure psychological distress (i.e., depression, anxiety, and stress), perceived stress, and subjective resilience. In addition, they were asked questions about mental health and psychosocial service acquisition.

**Results:**

The results indicate that levels of psychological distress were high among the target population; around 65% of respondents reported having some level of psychological distress. Moreover, women showed not only higher level of psychological distress but also revealed slightly lower subjective resilience as compared to male participants. Hierarchical regressions showed that subjective resilience significantly contributed to the predictive model of distress beyond demographics and having received or not mental health and psychosocial support. Subjective resilience was significantly associated to less anxiety (*R*^*2*^_adj_ = .157,* ΔR*^*2*^ = .022, *p* = .010) and stress (*R*^*2*^_adj_ = .083, *ΔR*^*2*^ = .026, *p* = .008) in Mam-Rashan camp; and to less depression (*R*^*2*^_adj_ = .184, *ΔR*^*2*^ = .095,* p* < .001), anxiety (*R*^*2*^_adj_ = .140, *ΔR*^*2*^ = .024, *p* = .034), stress (*R*^*2*^_adj_ = .046, *ΔR*^*2*^ = .047, *p* = .005), and perceived stress (*R*^*2*^_adj_ = .024, *ΔR*^*2*^ = .032, *p* = .022) in Shekhan camp.

**Conclusions:**

Conflict and displacement contribute to high level of psychological distress. Resilience, however, seem to have a negative association with psychological distress. Additionally, living conditions and sex also played an important role in both psychological distress and resilience. Consequently, Yazidi community residing in camps are in need of further support to alleviate the consequences of displacement. We critically discuss the differences in the results among participants per camp and by sex, and its implications.

## Introduction

Crisis and emergency situations resulting from conflict and natural and manmade disasters pose a significant risk to the affected population's psychosocial wellbeing. There are currently major refugee and displacement crises all over the world. The estimated 84 million people forcibly displaced by violence and conflicts—including 48 million internally displaced persons (IDPs)—amount to the highest recorded number in world history [1]. Regrettably, the number of people forced to flee their homes in search of safety is increasing, with citizens of Afghanistan, Ethiopia, and Ukraine being among the most recent examples [2, 3].

Because of the wars and conflicts that have ravaged the Middle East for decades, a substantial portion of displacement crises have occurred and are occurring in countries in this region. According to reports and data, in recent years, Iraq has the highest number of IDPs, ranking second only to Syria in 2014 [4, 5]. Iraq has a long history of war and violence, for decades, Iraqis have been subjected to repression and human rights violations, including torture and abuse. According to the Political Terror Scale [6], Iraq is one of the top five worst countries for human rights violations. As a result of their own governments’ and other external aggressiveness and violence, Iraqi citizens have been repeatedly forced to flee their homes in search of more secure living conditions and new sources of livelihood.

Recently, more than 3 million Iraqis have been displaced as a direct consequence of the Islamic State of Iraq and the Levant’s (ISIL's) aggression [7]. The United Nations declared a level three emergency in Iraq in June 2014 [8]. While the majority of the displaced population has returned to their home communities, the most recent figures reveal that approximately 1.2 million displaced Iraqis are still living in formal and informal shelters throughout Iraq [9, 10]. At the time of writing this paper, there were 26 camps, and 12 of them were located in the Duhok governorate. Despite the fact that the ISIL attacks have impacted the entire population of Iraq (including its multi-ethnic groups), Yazidis are among the most affected.

The Yazidis are an ethnoreligious, predominantly Kurdish-speaking group, and they reside mainly in Iraq. Yazidis have been the target of hatred for centuries and have faced the possibility of genocide many times over. The assault by ISIL provoked the displacement of more than 400,000 individuals from the Yazidi minority. Their villages and cities were destroyed and looted, and about 6000 girls and women were kidnapped [11, 12] and subjected to inhumane treatment (including being sexually assaulted, forced to change religion, and, in some cases, sold as sex objects). Those who could escape these barbaric attacks have been living in camps in the Kurdistan region of Iraq that were established to host internally displaced populations. The Mam-Rashan and Shekhan camps being one of the first established camps among many others, which house Yazidis, are where this study was conducted.

Internal displacement is defined as the compulsory movement of people due to internal conflicts that force people and their families to leave their original place of residency and move to another location that offers a greater degree of security and stability [13]. Displaced individuals may lose their homes and belongings, be separated from family and loved ones, lose community support, and witness violence, destruction, and death. General psychological distress and, in particular, posttraumatic stress disorder and depression have been long established as common mental health concerns amongst people who have fled their homes due to instability or obligatory migration [14]. These distressing events, which have been happening for more than four decades, have had tremendous psychological effects on Iraqis—especially amongst minority groups like Yazidis. Data suggests that almost all Yazidis whom ISIS formerly enslaved met the criteria for a probable DSM-5 PTSD diagnosis [15]. Further, Takin et al. [16] concluded that around 42.9% of Yazidis in their study met the DSM-5 diagnostic criteria for PTSD, 39.5% for major depression, and 26.4% for both disorders. Moreover, the psychological problems identified among Yazidis were not only PTSD but included diverse issues, including sleeping problems, depression, conversion disorder, and adjustment [17].

### Psychological impact of conflict and resilience among affected communities

The impact of conflict on psychological wellbeing is apparent. In its latest report, the WHO estimates that 22.1% of conflict-affected populations have mental disorders (e.g., depression, anxiety, posttraumatic stress disorder, bipolar disorder, and schizophrenia) at any point in time. The reported prevalence is 13.0% for mild forms and 5.1% for severe forms of the disorders mentioned [18]. The impact of violence on an individual’s wellbeing is indescribable in the sense that the negative effects of the conflict not only increase the level of mental health problems, abolish the normal societal support, and shake the family dynamics, they also have long-term impacts that can continue for subsequent generations [19, 20].

The Inter-Agency Standing Committee (IASC) guidelines, which are considered to be among the most respected in the humanitarian context refer to crises or emergencies as a "wide range of problems experienced at the individual, family, community, and societal levels. At every level, emergencies erode normally protective supports, increase the risks of diverse problems, and tend to amplify pre-existing problems of social injustice and inequality" [21, p. 6]. Crisis-affected populations face multi-layered challenges, from economic and security concerns to social and psychological problems.

To respond to the exacerbated needs of a population in crisis, governmental and nongovernmental organizations deploy resources through multidisciplinary approaches that can include provision of shelter, food, health, and mental health and psychosocial services. These responses aim to decrease the level of distress and help individuals cope with the difficulties they are experiencing. More importantly, community members create and locate new ways to resist the protracted effects of the crisis. Goodman et al. [22] argue that adversities can produce distinctive strengths that can be sources of resilience.

There is no doubt that experiences of trauma have negative psychological consequences. Furthermore, while the prevalence of mental health disorders is significantly higher among conflict-affected populations when compared to non-conflict-affected communities, it is vital to note that not all people in humanitarian crises suffer from psychological disorders [18, 21]. Therefore, the traditional interpretation of psychopathology of trauma incidents does not necessarily explain people’s personal experiences in a comprehensive manner. Bonanno et al. [23] suggest that most people respond to adversities with minimal disruptions in overall functioning. The phenomenon of not displaying psychopathology symptoms after a traumatic event does not qualify it as psychological numbness but rather as resilience [23].

Although resilience has been extensively studied, the term could convey different meanings based on the different scopes of the studies. Bonanno [24, p. 20] defines resilience as "the ability of adults in otherwise normal circumstances who are exposed to an isolated and potentially highly disruptive event, such as the death of a close relative or a violent or life-threatening situation, to maintain relatively stable, healthy levels of psychological and physical functioning."

The threshold of resilience varies depending on the severity of incidents and prior experiences [25], social and cultural context [26], and personality type [27]. The association between resilience and psychological wellbeing is well established [26, 28]. Furthermore, researchers [see, for example, 22, 23, 24] argue that resilient individuals can cope with traumatic events with no noticeable disruption in their ability to function. Resilience can also act as a protective factor in the prevention of developing psychological distress.

### Present research

The consequences of wars on populations and, more specifically, the psychological impacts of conflicts are appalling [18]. Generally, the accounts of human as well as material loss during war are documented. Nevertheless, besides some media attention (e.g., news articles and short reports), the recent tragedy experienced by the Yazidis in Iraq did not receive significant academic and scholarly consideration. In this study, the intention is to investigate the psychological status of Yazidis 6 years after the attack by ISIL. It is evidence-based that displacement causes visible loss, including homes, valuables, and belongings), but displacement also creates invisible psychological wounds. The data indicate that forced displacement is a significant risk factor and that it is directly linked to an increase in psychological disorders [see, for example, 18, 21, 29, 30, 31].

The purpose of the current study was threefold. We aimed to explore and compare the state of psychological distress and the sense of resilience among displaced Yazidis in two IDP camps with different characteristics: Mam-Rashan and Shekhan camps. We analyzed the sociodemographic components of the two camps and compared the levels of psychological distress and resilience between both. The characteristics of and available services for each camp are different; for example, Mam-Rashan camp has more adequate shelter, a larger living space, and more abundant community services than Shekhan camp, which is located nearer to Shekhan town and thus has greater access to services and commodities in the nearby community. However, the services inside the Shekhan camp itself are minimal. We might expect that the level of psychological distress at this camp could be high given the trauma experienced and their current living situation combined. As indicated by Mels et al. [32], people who are displaced reveal higher levels of psychological distress.

We also were interested in analysing the gender differences of psychological distress and the sense of resilience of IDP Yazidis in both camps. Yazidi women and girls had not only been targeted by ISIL but were also bearing the consequences of the displacement at large. These include many potential changes, such as the alteration of their role in the absence of a breadwinner, tormenting household needs, an ambiguous future, and disruptive family dynamics. Given the strong link between displacement and the exacerbation of existing emotional health challenges among displaced women [33–35], we expected that male and female participants would score significantly differently, with women displaying higher psychological distress (Hypothesis 1).

Finally, we intended to investigate the contributing factor of subjective resilience in psychological distress in both camps beyond demographics differences and the psychosocial mental support received. Bonanno [24] suggests that during the course of their lives, many people are exposed to potential traumatic incidents. While people react differently, most individuals manage to endure temporary loss or traumatic events remarkably well. We anticipated that subjective resilience could be a contributing factor in understanding psychological distress beyond the sex of participants and other sociodemographic aspects, such as marital status, parenthood, and access to mental health and psychosocial support (Hypothesis 2).

In this study, we have presented information to demonstrate that resilience and psychological distress have been studied quite extensively in contexts of developed countries but less so in humanitarian emergency settings. Attention on the prevalence of mental disorders or the significance of mental health is not lacking in mainstream research. Rather, the gap lies in the lack of focus on humanitarian settings and specifically on the impact of the ISIL attack on the psychological wellbeing of Yazidis living in camps. As far as our knowledge goes, the tragedy experienced by the Yazidis in Iraq has not received significant academic and scholarly consideration. A deeper comprehension of their level of psychological distress and resilience, as well as the situational factors and sociodemographic characteristics, especially related to gender, associated to psychological distress and resilience might be of great utility not only to improve our knowledge about the factors associated with psychological wellbeing under difficult experiences, but to re-define actions to contribute to psychological adjustment in a context of displacement.

## Method

### Participants

The population of interest for this research is IDPs coming from the Sinjar area who settled in two camps, Mam-Rashan and Shekhan, which are located in the Duhok governorate in the Kurdistan region of Iraq. We have selected both camps for different reasons. Firstly, both camps are majority Yazidis compared to some others that are mixed. Secondly, both camps are among the very first established camps after the attack of ISIS. And lastly, the structure of these camps is different, which was of our interest as we wanted to explore its association with psychological distress and subjective resilience.

Each participant came from a conflict-affected area and was displaced from their place of origin as a result of the 2014 ISIL attacks in Iraq. At the time of data collection, the total population in both camps was 10,411 people, with a potential 5926 adults (i.e., 18 years or older) to be considered in this study. A systematic sampling method was used to select the number of tents/cabins in chronological order following the design of the camp to cover the whole population with a representative sample. Concerning the number of respondents, 442 people voluntarily agreed to participate in the study, constituting a representative sample with a margin of error of 4.48%. We omitted seven respondents with incomplete data and 13 participants who identified themselves as Muslims because the study is focused on Yazidis. The final sample was comprised of 422 participants (50.9% female) from 18 to 77 years old (*M*_*age*_ = 32.63, *SD* = 13.01). Sensitivity analyses conducted with G*Power [36] demonstrated that Sensitivity analyses conducted with G*Power demonstrated that a sample of 422 participants could detect an effect size of *f* = .137 (η^2^ = .018) in an ANOVA (fixed effects, special, main effects, and interactions) with four groups (two camps by sex). A linear multiple regression (fixed model, R^2^ increase) with a probability of α error of .05 and a power (1–β error probability) of .80 for one tested predictor and seven predictors can detect an *f* ^2^ of .030 in the sample of Mam-Rashan (n = 257) and of .048 for the sample of Shekahn camp (n = 165).

### Variables and instruments

We employed face-to-face interviews to administer the questionnaire to each participant to inquire about their psychological distress, perceived stress, subjective resilience, and MHPSS.

#### Psychological distress

Participants’ psychological distress was measured using the Depression, Anxiety and Stress Scale—21 Items [DASS-21, 37], through which states of depression, anxiety, and stress are measured. The DASS-21 is composed of 21 items with three subscales. The depression subscale contains seven items to assess dysphoria, hopelessness, devaluation of life, self-deprecation, lack of interest/involvement, anhedonia, and inertia (α = .68). The anxiety dimension contains seven items to measure autonomic arousal, skeletal muscle effects, situational anxiety, and the subjective experience of anxious affect (α = .76). The stress subscale has seven items to assess difficulty relaxing, nervous arousal, and one’s experience of being easily upset/agitated, irritable/overreactive, and impatient (α = .79). Participants answered using a 4-point Likert scale from 0 (*does not apply at all*) to 3 (*applies most of the time*). Scores for the depression, anxiety, and stress subscales are calculated separately by summing the scores for the relevant seven items and multiplying them by 2 to calculate the final score, according to the instructions (see Lovibond and Lovibond [37]). The interpretation scores are divided into five categories: *normal* state (scores between 0–9 depression, 0–7 anxiety, and 0–14 stress), *mild* psychological symptoms (scores between 10–13 depression, 8–9 anxiety, and 15–18 stress), *moderate* psychological distress (14–20 depression, 10–14 anxiety, and 19–25 stress), *severe* symptoms (scores between 21–27 for depression, 15–19 for anxiety, and 26–33 for stress), and *extremely severe* symptoms (scores of 28 and above for depression, 20 and above for anxiety, and 34 and above for stress).

#### Perceived stress

The Perceived Stress Scale [38] has 14 items that are used to measure the degree to which one appraises the situations in their life as stressful. Respondents were asked to rate the frequency of their feelings and thoughts related to events and situations that had occurred in the last month. Participants answered using a 5-point Likert scale from 0 (*never*) to 4 (*very often*). When all the items were summed up, the scores ranged from 0 to 56. A higher score indicates greater stress. Seven of the 14 items were inverted (α = .77).

#### Resilience

This variable was measured with the Brief Resilience Scale [39]. Participants were asked to what extent they agreed with six items that relate to their ability to stand and face difficult times through use of a Likert scale (from 1 = *strongly disagree* to 5 = *strongly agree*). These six items are as follows: (a) “I tend to bounce back quickly after hard times,” (b) “I tend to recover quite fast from stressful events,” (c) “I usually come through difficult times with little trouble,” (d) “I have a hard time making it through stressful events,” (e) “It is hard for me to snap back when something bad happens,” and (f) “I tend to take a long time to get over setbacks in my life” (the last three reversed, α = .71).

#### Mental health and psychosocial support

Participants were asked to answer the following four questions in order to explore the availability, type, and efficacy of MHPSS services at the targeted camps: (a) "Have you ever received MHPSS services since your arrival to camp?" (Yes/No), (b) "What type of MHPSS services (e.g., individual, support groups, vocational training)?", (c) “How long was the duration of the provided MHPSS support (e.g., single session, two to four sessions, five to seven sessions, or more than seven sessions)?”, and (d) "Was the MHPSS intervention useful?" (Yes/No). We explained the MHPSS services term and described types of such services before asking MHPSS-related questions to avoid using ambiguous language.

#### Demographic information

Participants reported their sex, age, civil state, educational level, employment status, and religion.

### Procedure

The previously outlined scales and questions were included in a questionnaire that we administered through face-to-face interviews. Since Yazidi community speak in Kurdish (Kurmanji dialect) and some members speak in Arabic, we translated the questionnaire into both Arabic and Kurdish Kurmanji dialect. Further, all people involved in the data collection were graduate psychologist and were able to speak both Arabic and Kurdish. Participation was voluntary, and no economic compensation was offered. Psychological services were available for each participant if they desired a debriefing to regulate emotions and normalize any feelings raised during the interview. The study’s objectives and duration were explained to participants, and their consent was then obtained. Prior to the data collection, a research proposal was submitted to the University of Duhok (Iraq) for their approval. In addition, we submitted a letter of access (to collect the data) to the camps’ administrative authorities.

## Results

### Demographic profile per camp

As presented in Table 1, most participants living in both camps were unemployed. Compared to Mam-Rashan, Shekhan camp revealed a more vulnerable demographic profile: most IDP participants in Shekhan camp were women and had children. This camp also presented a higher proportion of illiteracy (when compared with Mam-Rashan camp). A higher proportion of participants in Mam-Rashan were single.

**Table 1:** Sociodemographic profile of participants per camp

Sociodemographic variables	Camps	
	Mam-Rashan (n = 257)	Shekhan (n = 165)
*Age*		
Mean	29.98	35.75
SD	12.20	13.20
*Sex*		
Male	63.4%	26.7%
Female	36.6%	73.3%
*Marital status*		
Single	41.5%	17%
Married	58.8%	75.2%
Divorced	N/A	1.8%
Widowed	N/A	6.1%
*Children*		
Yes	51.4%	73.9%
No	48.6%	26.1%
*Education*		
Illiterate	23.3%	56.4%
Primary school	19.1%	23%
Secondary school	47.5%	15.2%
Undergraduate	10.1%	5.5%
*Employment status*		
Unemployed	84.8%	82.4%
Employed	15.2%	17.6%

Among all participants, 78.2% had not received any MHPSS services, whereas only 21.8% of respondents (15.6% from Mam-Rashan camp and 31.5% from Shekhan camp) had received some MHPSS services in the last 6 six years. Of these participants, 40.2% had received individual sessions (i.e., one-on-one sessions with a mental health professional), 37% had attended group support (i.e., a group of participants attending a series of psychosocial support sessions), 20.7% had participated in vocational training (i.e., training activities that aim to equip participants with skills and knowledge concerning specific trade- or occupation-related subjects, including sewing, cooking, mechanics, and so on), and 2.2% had received psychiatric care (i.e., services where psychotropic medications were prescribed by a psychiatrist or trained medical staff). The duration of MHPSS varied depending on the type of service provided. While the vocational training and group support cycle lasted for nine sessions, individual sessions typically occurred over three sessions. Most participants who had received MHPSS services (87%) identified the provided service as helpful.

### Psychological distress and subjective resilience

**Fig. 1:**
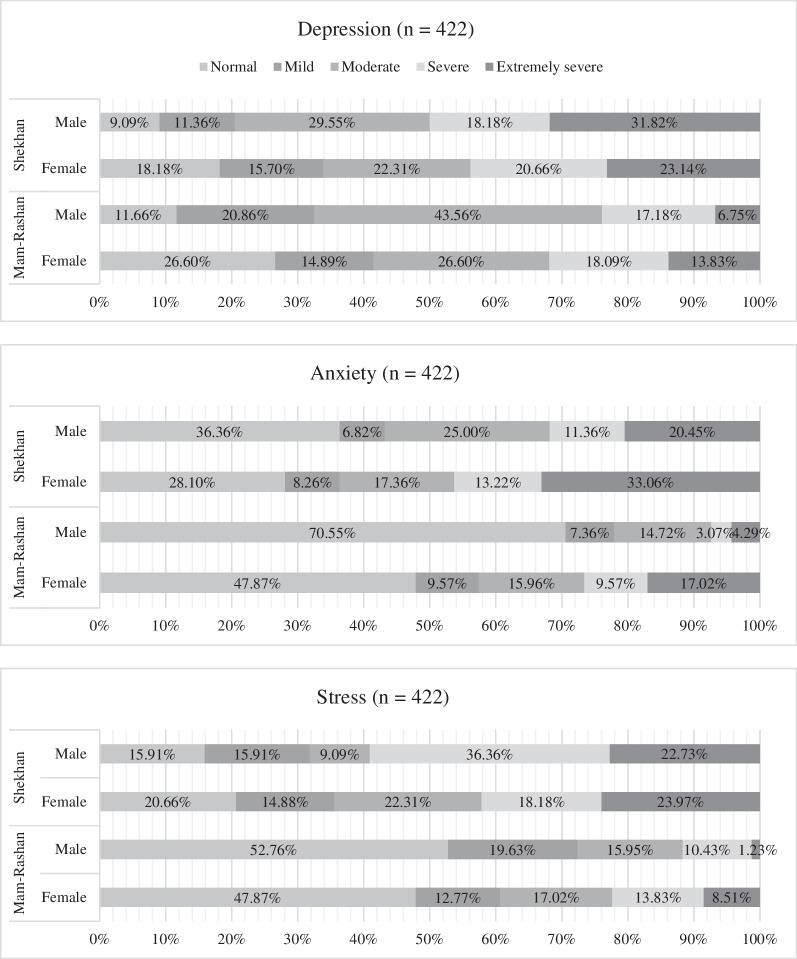
Level of psychological distress of women and men by camp

Generally, the results indicate high levels of psychological distress among participants. Taken together, a majority of respondents (65%) reported having some level of psychological distress. In examining the results more closely, 14% of respondents had mild, 22% had moderate, 14% had severe, and 15% had extremely severe levels of psychological distress. Detailed results of these distinct levels of psychological distress by sex and by camp are presented in Fig. 1.

To test whether psychological distress, perceived stress, and resilience varied depending on the camp and the sex of the participants, a two-factor ANOVA was conducted with sex and camp as factors. Results reveal a multivariate effect of camp, Wilk's Λ = .81, *F*(5, 414) = 19.68, *p* < .001, η^2^_p_ = .192. There was a univariate effect of camp on depression, *F*(1418) = 11.43, *p* < .001, η^2^_p_ = .027; anxiety, *F*(1418) = 16.74, *p* < .001, η^2^_p_ = .039; stress, *F*(1418) = 52.27, *p* < .001, η^2^_p_ = .111; perceived stress, *F*(1418) = 45.73, *p* < .001, η^2^_p_ = .099; and resilience, *F*(1418) = 18.07, *p* < .001, η^2^_p_ = .041. As shown in Table 2, participants who lived in Shekhan camp revealed higher level of psychological distress (depression, anxiety, stress and perceived stress) compared to participants from Mam-Rashan camp. However, residents of Shekhan camp reported a higher level of subjective resilience when compared with the respondents of Mam-Rashan camp.

**Table 2:** Comparisons between camps on psychological distress and subjective resilience

Location_ IDP camp	*M*	*SE*	95% Confidence Interval	
			Low	High
*Depression*				
Mam Rashan	16.26_b_	0.56	15.16	17.36
Shekhan	19.45_a_	0.76	17.96	20.95
*Anxiety*				
Mam Rashan	8.45_b_	0.54	7.39	9.51
Shekhan	12.17_a_	0.73	10.73	13.61
*Stress*				
Mam Rashan	16.34_b_	0.60	15.17	17.52
Shekhan	23.63_a_	0.81	22.04	25.23
*Perceived stress*				
Mam Rashan	23.68_b_	0.47	22.75	24.61
Shekhan	29.08_a_	0.64	27.81	30.34
*Subjective resilience*				
Mam Rashan	18.95_b_	0.29	18.38	19.52
Shekhan	21.04_a_	0.39	20.26	21.81

Estimated marginal means. Subscripts (a,b) refer to pairwise comparisons with Bonferroni tests by camp. Subscripts “a” represent higher values, whereas subscripts “b” represent lower values

Sex also had a multivariate effect on psychological distress, Wilk's Λ = .83, *F*(5414) = 16.83, *p* < .001, η^2^_p_ = .169. Results demonstrate a univariate effect of sex on anxiety, *F*(1418) = 58.96, *p* < .001, η^2^_p_ = .124; stress, *F*(1418) = 5.07, *p* = .025, η^2^_p_ = .012; and resilience, *F*(1418) = 9.15, *p* = .003, η^2^_p_ = .021. As presented in Table 3, the female participants reported considerably higher levels of psychological distress (anxiety and stress) than male participants and expressed a lower sense of resilience when compared with male respondents. However, sex had no univariate effects on depression, *F*(1418) = .01, *p* = .979, η^2^_p_ < .001 or on perceived stress, *F*(1418) = 2.28, *p* = .132, η^2^_p_ = .005.

**Table 3:** Comparisons between women and men on psychological distress and subjective resilience

	*M*	*SE*	95% Confidence interval	
			Low	High
*Depression*				
Women	17.87_a_	0.59	16.70	19.04
Men	17.84_a_	0.73	16.40	19.29
*Anxiety*				
Women	13.80_a_	0.57	12.68	14.92
Men	6.82_b_	0.71	5.43	8.21
*Stress*				
Women	21.12_a_	0.63	19.88	22.37
Men	18.85_b_	0.78	17.31	20.39
*Perceived stress*				
Women	26.98_a_	0.50	26.00	27.97
Men	25.78_a_	0.62	24.56	27.00
*Subjective resilience*				
Women	19.25_b_	0.31	18.65	19.86
Men	20.73_a_	0.38	19.99	21.48

Estimated marginal means. Subscripts (a,b) refer to pairwise comparisons with Bonferroni tests by sex. Subscripts “a” represent higher values, whereas subscripts “b” represent lower values

The inclusion of demographic variables (e.g., civil state, parenthood, educational level, and employment) as covariates did not alter the results except concerning the effect of camp on depression, which lost its significance once demographics were considered, *F*(1414) = 1.83, *p* = .176, η^2^_p_ = .004.

To summarize, psychological distress (especially anxiety and stress) was reported as being higher among women, particularly in Shekhan camp. As presented in Table 4, the level of anxiety reported by women is double the reported level among men in both camps, with exceptionally higher levels in Shekhan camp. However, the level of depression was not significantly different between men and women in either camp. Similarly, the level of perceived stress in Shekhan camp was not significantly different between male and female participants. However, there were neither multivariate nor univariate two-way interaction effects between camp and sex on the variables, *ps* > .077.

**Table 4:** General (regardless of the level of) psychological distress, perceived stress, and resilience reported by men and women in both camps

Location_ IDP camp	Women				Men			
	*M*	*SE*	95% Confidence Interval		*M*	*SE*	95% Confidence Interval	
			Low	High			Low	High
*Depression*								
Mam Rashan	16.15_b_	0.89	14.40	17.90	16.37_b_	0.68	15.04	17.70
Shekhan	19.59_a_	0.79	18.04	21.13	19.32_a_	1.30	16.76	21.88
*Anxiety*								
Mam Rashan	11.17_b_	0.86	9.48	12.86	5.73_c_	0.65	4.45	7.01
Shekhan	16.43_a_	0.76	14.94	17.92	7.91_c_	1.25	5.44	10.37
*Stress*								
Mam Rashan	17.62_b_	0.95	15.75	19.49	15.07_c_	0.72	13.65	16.49
Shekhan	24.63_a_	0.84	22.98	26.28	22.64_a_	1.39	19.90	25.37
*Perceived stress*								
Mam Rashan	24.99_b_	0.75	23.51	26.47	22.37_c_	0.57	21.25	23.50
Shekhan	28.98_a_	0.66	27.67	30.28	29.18_a_	1.10	27.02	31.35
*Subjective resilience*								
Mam Rashan	18.39_c_	0.46	17.49	19.30	19.52_c_	0.35	18.83	20.20
Shekhan	20.12_b_	0.41	19.32	20.92	21.95_a_	0.68	20.63	23.28

Estimated marginal means. Subscripts (a,b,c) refer to pairwise comparisons with Bonferroni tests. Horizontal comparisons compare women with men, whereas vertical comparisons compare camps. Subscripts “a” represent the highest values, whereas subscripts “c” represent the lowest values

### The relationship between resilience and psychological distress

Means, standard deviations, and correlation coefficients between resilience and psychological distress are presented in Table 5. The results reveal that resilience has a significant negative relationship with depression, anxiety, and stress in both camps. Although perceived stress is not significantly associated with resilience in Mam-Rashan camp, it has a significant negative relationship with resilience in Shekhan camp. These results mean that, the more resilient the person reported themself to be, the less depression, anxiety, and stress they reported in both camps and the less perceived stress they reported in Shekhan camp.

**Table 5:** Descriptive statistics and partial correlations controlled by sex in both camps

Camps	Variables	*M*	*SD*	1	2	3	4	5
Mam-Rashan	1. Depression	16.05	7.82	–				
	2. Anxiety	7.53	7.71	.51^**^	–			
	3. Stress	15.81	8.66	.62^**^	.61^**^	–		
	4. Perceived stress	23.27	7.66	.55^**^	.42^**^	.48^**^	–	
	5. Resilience	19.17	3.97	− .12	− .14^*^	− .15^*^	.09	–
Shekhan	1. Depression	19.51	9.77	–				
	2. Anxiety	14.15	10.30	.46^**^	–			
	3. Stress	24.09	10.09	.69^**^	.51^**^	–		
	4. Perceived stress	29.03	6.91	.54^**^	.24^**^	.55^**^	–	
	5. Resilience	20.61	5.28	− .31^**^	− .17^*^	− .21^*^	–.17^*^	-

**p < .001, *p < .05

A hierarchical multiple regression analysis was calculated to assess how the presence of resilience can predict the level of psychological distress (e.g., depression, anxiety, and stress) and perceived stress after controlling for the influence of the demographic variables and MHPSS services separately per camp. First, we conducted the analyses for Mam-Rashan camp by entering the demographics at Step 1, the MHPSS services at Step 2, and the subjective resilience at Step 3. As presented in Table 6, the results showed that subjective resilience significantly contributed to the predictive model of distress beyond demographics and whether or not MHPSS services were received. Subjective resilience was significantly associated to less anxiety (*R*^2^_adj_ = .157, Δ*R*^2^ = .022, *p* = .010) and less stress (*R*^2^_adj_ = .083, Δ*R*^2^ = .026, *p* = .008) among IDPs in Mam-Rashan camp. The inclusion of age as a covariate in the model did not alter the results and was not significantly associated to distress.

**Table 6:** Hierarchical regressions of demographics, mental health and psychosocial services, and resilience as predictors of psychological distress in Mam-Rashan camp

		Depression			Anxiety			Stress			Perceived stress		
		*B*	*β*	*p*	*B*	*β*	*p*	*B*	*β*	*p*	*B*	*β*	*p*
1	Intercept	16.43		< .001	11.16		< .001	16.78		< .001	24.51		< .001
	Sex	0.26	.02	.798	**− 5.74**	**− .35**	** < .001**	**− 2.91**	**− .16**	**.012**	**− 3.10**	**− .20**	**.002**
	Marital status	2.46	.16	.179	2.15	.14	.220	3.45	.20	.094	2.43	.16	.171
	Children	− 0.08	− .01	.967	− 1.08	− .07	.540	− 1.43	− .08	.488	− 0.49	− .03	.782
	Education	**− 1.27**	**− .16**	**.029**	− 0.65	− .08	.244	− 0.37	− .04	.564	− 0.64	− .08	.252
	Employment	0.74	.03	.595	**2.77**	**.13**	**.037**	2.06	.09	.186	**3.55**	**.17**	**.008**
		*F*(5251) = 3.61, *p* = .004, *R*^*2*^_adj_ = .048			*F*(5251) = 8.61, *p* < .001, *R*^*2*^_adj_ = .129			*F*(5251) = 2.75, *p* < .019, *R*^*2*^_adj_ = .033			*F*(5251) = 4.82, *p* < .001, *R*^*2*^_adj_ = .069		
2	Intercept	16.49		< .001	11.03		< .001	16.68		< .001	24.64		< .001
	Sex	0.22	.01	.833	**− 5.65**	**− .35**	** < .001**	**− 2.84**	**− .16**	**.014**	**− 3.19**	**− .20**	**.001**
	Marital status	2.58	.16	.162	1.94	.12	.268	3.29	.19	.112	2.66	.17	.132
	Children	− 0.08	− .01	.966	− 1.07	− .07	.540	− 1.43	− .08	.489	− 0.50	− .03	.779
	Education	**− 1.20**	**− .15**	**.040**	− 0.77	− .09	.165	− 0.47	− .05	.469	− 0.50	− .06	.369
	Employment	0.67	.03	.631	**2.91**	**.13**	**.028**	2.16	.09	.165	**3.41**	**.16**	**.011**
	Received MHPSS services	− 1.19	− .06	.371	2.28	.11	.073	1.77	.07	.236	− 2.46	− .12	.054
		*F*(6250) = 3.14, *p* = .006, *R*^*2*^_adj_ = .048*,* Δ*R*^*2*^ = .003*, p* = .371			*F*(6250) = 7.78, *p* < .001, *R*^*2*^_adj_ = .137*,* Δ*R*^*2*^ = .011*, p* = .073			*F*(6250) = 2.53, *p* < .021, *R*^*2*^_adj_ = .035*,* Δ*R*^*2*^ = .005*, p* = .236			*F*(6250) = 4.69, *p* < .001, *R*^*2*^_adj_ = .080, Δ*R*^*2*^ = .013*, p* = .054		
3	Intercept	20.33		< .001	16.63		< .001	23.43		< .001	21.85		< .001
	Sex	0.38	.02	.710	**− 5.41**	**− .33**	** < .001**	**− 2.55**	**− .14**	**.026**	**− 3.31**	**− .21**	**.001**
	Marital status	2.52	.16	.169	1.86	.12	.283	3.19	.18	.118	2.70	.17	.126
	Children	− 0.13	− .01	.942	− 1.15	− .07	.505	− 1.53	− .09	.454	− 0.45	− .03	.796
	Education	**− 1.15**	**− .14**	**.048**	− 0.70	− .09	.203	− 0.39	− .04	.550	− 0.54	− .07	.336
	Employment	1.14	.05	.417	**3.60**	**.17**	**.007**	3.00	.12	.056	**3.06**	**.14**	**.025**
	Received MHPSS services	− 1.22	− .06	.358	2.24	.10	.075	1.72	.07	.243	− 2.45	− .12	.055
	Resilience	− 0.21	− .11	.093	**− 0.31**	**− .16**	**.010**	**− 0.37**	**− .17**	**.008**	0.15	.08	.204
		*F*(7249) = 3.12, *p* = .004, *R*^*2*^_adj_ = .055, Δ*R*^*2*^ = .011*, p* = .093			*F*(7249) = 7.79, *p* < .001, *R*^*2*^_adj_ = .157, Δ*R*^*2*^ = .022*, p* = .010			*F*(7249) = 3.24, *p* < .003, *R*^*2*^_adj_ = .083*,* Δ*R*^*2*^ = .026*, p* = .008			*F*(7249) = 4.26, *p* < .001, *R*^*2*^_adj_ = .082*,* Δ*R*^*2*^ = .006,* p* = .204		

Statistically significant predictors and values are showed in bold

We conducted a hierarchical regression analysis among participants from Shekhan camp to test the hypothesis that resilience predicts psychological distress beyond demographics and access to MHPSS services. The detailed results are presented in Table 7. In all three models, we controlled for sex, marital status, children, education, and employment. We added MHPSS services in Step 2 and subjective resilience in Step 3. The results demonstrate that depression (*R*^2^_adj_ = .184, Δ*R*^2^ = .095, *p* < .001), anxiety (*R*^2^_adj_ = .140, Δ*R*^2^ = .024, *p* = .034), stress (*R*^2^_adj_ = .046, Δ*R*^2^ = .047, *p* = .005), and perceived stress (*R*^2^_adj_ = .024, Δ*R*^2^ = .032, *p* = .022) had a statistically significant negative association with subjective resilience once controlled for demographics and MHPSS services in Shekhan camp. In other words, the results indicate that a sense of resilience was associated with less depression, anxiety, stress and perceived stress among Yazidi participants. Interestingly, subjective resilience was more strongly associated with psychological distress in the camp with a worst psychological adjustment, although no causal assumptions can be established. The inclusion of age as a covariate in the model did not alter the results and was not significantly associated to distress.

**Table 7:** Hierarchical regressions of demographics, mental health and psychosocial services, and resilience as predictors of psychological distress in Shekhan Camp

	Steps and variables	Depression			Anxiety			Stress			Perceived stress		
		*B*	*β*	*p*	*B*	*β*	*p*	*B*	*β*	*p*	*B*	*β*	*p*
1	Intercept	15.62		< .001	14.76		< .001	22.95		< .001	27.55		< .001
	Sex	1.39	.06	.461	**− 7.77**	**− .34**	** < .001**	− 1.45	− .06	.475	0.22	.01	.877
	Marital status	**4.34**	**.29**	**.001**	1.97	.13	.136	2.58	.17	.061	1.38	.13	.147
	Children	0.26	.01	.896	− 0.29	− .02	.887	− 1.10	− .05	609	− .007	− .004	.963
	Education	− 1.16	− .11	.236	0.04	< .001	.966	− 0.56	− .05	.595	0.33	.04	.651
	Employment	− 0.37	− .02	.854	− 1.43	− .05	.495	1.35	.05	.538	0.02	.001	.988
		*F*(5159) = 4.43, *p* = .001, *R*^*2*^_adj_ = .095			*F*(5159) = 5.67, *p* < .001, *R*^*2*^_adj_ = .125			*F*(5159) = 1.29, *p* = .271, *R*^*2*^_adj_ = .009			*F*(5159) = 0.47, *p* = .800, *R*^*2*^_adj_ = − .017		
2	Intercept	16.16		< .001	15.28		< .001	22.61		< .001	28.57		< .001
	Sex	0.99	.05	.620	**− 8.16**	**− .35**	** < .001**	− 1.20	− .05	.577	− 0.62	− .04	.676
	Marital status	**4.19**	**.28**	**.001**	1.82	.12	.174	2.67	.17	.057	1.07	.10	.264
	Children	0.25	.01	.901	− 0.30	− .01	.883	− 1.09	− .05	.612	− 0.09	− .01	.950
	Education	− 1.13	− .11	.249	0.07	.01	.944	− 0.58	− .05	.585	0.39	.05	.591
	Employment	− 0.16	− .01	.939	− 1.23	− .05	.566	1.21	.05	.587	0.47	.03	.756
	Received MHPSS services	− 1.07	− .05	.528	− 1.03	− .05	.557	0.67	.03	.713	− 2.23	− .15	.078
		*F*(6158) = 3.75, *p* = .002, *R*^*2*^_adj_ = .091, Δ*R*^*2*^ = .002, *p* = .528			*F*(6158) = 4.77, *p* < .001, *R*^*2*^_adj_ = .121, Δ*R*^*2*^ = .002*, p* = .557			*F*(6158) = 1.09, *p* = .370, *R*^*2*^_adj_ = .003, Δ*R*^*2*^ = .001, *p* = .713			*F*(6158) = 0.92, *p* = .482, *R*^*2*^_adj_ = − .003*,* Δ*R*^*2*^ = *− *019, *p* = .078		
3	Intercept	29.01		< .001	22.10		< .001	31.95		< .001	33.83		< .001
	Sex	1.80	.08	.342	**− 7.73**	− .33	** < .001**	− 0.61	− .03	.774	− 0.29	− .02	.846
	Marital status	**3.91**	**.26**	**.002**	1.67	.11	.207	2.47	.16	.073	0.96	.09	.312
	Children	− 0.47	− .02	.802	− 0.69	− .03	.737	− 1.61	− .07	.446	− 0.39	− .03	.791
	Education	− 1.48	− .14	.115	− 0.11	− .01	.912	− 0.83	− .08	.427	0.25	.03	.727
	Employment	1.06	.04	.589	− 0.58	− .02	.786	2.10	.08	.341	0.97	.05	.523
	Received MHPSS services	− 1.63	− .08	.310	− 1.33	− .06	.445	0.26	.01	.884	**− 2.46**	**− .17**	**.049**
	Resilience	**− 0.59**	**− .32**	** < .001**	**− 0.31**	**− .16**	**.034**	**− 0.43**	**− .22**	**.005**	**− 0.24**	**− .18**	**.022**
		*F*(7157) = 6.29, *p* < .001, *R*^*2*^_adj_ = .184*,* Δ*R*^*2*^ = .095*, p* < .001			*F*(7157) = 4.83, *p* < .001, *R*^*2*^_adj_ = .140, Δ*R*^*2*^ = .024, *p* = .034			*F*(7157) = 2.12, *p* = .044, *R*^*2*^_adj_ = .046, Δ*R*^*2*^ = .047, *p* = .005			*F*(7157) = 1.57, *p* = .146, *R*^*2*^_adj_ = .024, Δ*R*^*2*^ = .032, *p* = .022		

Statistically significant predictors and values are showed in bold

## Discussion

The humanitarian crisis created by ISIL has convulsed the world and dramatically impacted the entire population of Iraq, especially Yazidis. In this study, we explored the level of psychological distress and resilience among Yazidis in two camps in the Kurdistan region of Iraq: Mam-Rashan and Shekhan. We also examined the association between resilience and psychological distress beyond the influence of demographics and MHPSS service acquisition.

The results reveal that participants have experienced a high level of psychological distress, including depression, anxiety, stress, and perceived stress. These results confirm the already established hypothesis that conflict-affected populations endure more significant psychological problems than people who have not been impacted by such hostilities [14, 18]. However, they contrast with the notion that time heals all wounds. Six years after the ISIL attack, participants have maintained a high level of psychological distress. This could also be due to the daily stressors (e.g., poverty, social isolation, and poor or insecure neighbourhoods) that are proven to worsen psychological distress [21]. Nevertheless, it is also argued that an affected population bears the burden of the traumatic experiences years after the incident [19]. The mental health and psychosocial problems of displaced people are inextricably linked, necessitating multiple supports to sustain the recovery journey. The IASC [21] argues that people who have experienced a humanitarian crisis are affected differently from those who have not. Thus, they require different kinds of support. It further advocates for the emergency responders to develop multilayered support that meets the needs of different groups.

In the current study, female participants reported higher levels of psychological distress (in the form of anxiety and stress) and less subjective resilience than their male counterparts. This result is consistent with previous research indicating that women are more prone to psychological distress during displacement [32, 40, 41]. Such consequences could be the result of violence, including harassment by armed groups [42]. Furthermore, the burden of becoming head of the household or a mother at a young age, which frequently occurs in armed conflicts, could negatively contribute to higher psychological distress [43]. Women are commonly victims of sexual violence and trafficking [44]. In addition, women often become war tools used by armed militias to demoralize enemies and display power and dominance [45]. The data demonstrate that sex plays a significant role in resilience, and it has been reported that men have a higher level of resilience than females [46, 47].

While participants are originally from the same location, at the time of this study, they have been living in two different camps. Results demonstrate that participants living in Shekhan camp presented a higher level of psychological distress when compared with respondents from Mam-Rashan camp. Contributors such as living conditions, available services, and space given to displaced people in each camp might have influenced the status of their psychological distress and resilience. The IDPs in Shekhan camp have less services and a lower quality of living conditions (e.g., people living in tents in Shekhan camp versus cabins in Mam-Rashan camp). They also have less space around their tents compared to the IDPs at Mam-Rashan, where each cabin sits on a small space of land. Among other factors, access to services and psychosocial supports as well as quality living conditions are associated with more beneficial psychological wellbeing [21, 48, 49]. Furthermore, the number of participants from Shekhan camp who reported having children is considerably higher than in Mam-Rashan. Bird [50] argues that parents who have children report higher levels of psychological distress than those without a child. In addition, the results reveal a higher number of married people residing in Shekhan camp. While being married is considered a protective factor for wellbeing, not all marriage circumstances can positively contribute to psychological wellbeing [51]. Data show that levels of psychological distress are higher among dysfunctional families [52]. Cotten [52] further argues that typical family support is not present in a dysfunctional family dynamic. Moreover, displacement and scarcity of resources are expected to negatively impact the family dynamic among the Yazidi population. Despite the more vulnerable profile of Shekhan camp, higher subjective resilience was reported among their inhabitants. In this regard, Richardson [53] argues that people achieve growth and adaption through disruption.

Regarding the relationship between psychological distress and resilience, the results indicate that there was a negative and significant correlation between both variables in both camps. The more resilience one person exhibited, the less psychological distress they indicated, even when sex was controlled for. This result confirms the significant role of resilience in addressing psychological distress for an individual who has been exposed to adversities [see, for example, 24]. Researchers also suggest that positive coping strategies could substantially contribute to a more positive mental health outcome [54]. It should be noted that participants reported their belief of being resilient, that is, experiencing a form of subjective or perceived resilience. The more resilient participants considered themselves to be, the less psychological distress they reported. Due to the cross-sectional design of this study, directionality cannot be confirmed. Only a negative relationship between resilience and the psychological distress reported was established.

The hierarchical multiple regression analysis revealed that resilience can significantly improve the predictive model of psychological distress (e.g., depression, anxiety, stress, and perceived stress), especially in Shekhan camp. The results demonstrate that the proposed relationship between resilience and psychological distress is supported beyond the influence of demographics and MHPSS service acquisition for both camps. The participants from conflict-affected areas who reported a higher level of resilience communicated a lower level of psychological distress [55, 56]. It is likely that having strength and resources to combat the daily stressors contributes to personal resilience, which in turn might act as a protective factor. Despite that emergencies and conflicts can be distressing for many people, there are personal and collective sources of psychosocial resilience “(i.e., psychosocial resilience describes people’s ability to cope with stress in two forms; personal resilience, describes how particular people respond to the challenges they face. And second is collective resilience of how people collectively respond to, cope with, and recover from emergencies)” [57]. Additionally, Bonanno et al. [24] and Goodman et al. [22] suggest that resilience can act as a buffer in preventing the progression of psychological problems.

While psychological disorders and their prevalence have been studied in armed conflict and non-affected conflict contexts, neither the Yazidi community nor the consequences of the ISIL attack on Iraqis has received adequate academic attention. In this study, we explored the psychological distress and resilience among the minority Yazidi group in Iraq: a group and a country that have encountered decades of conflict and persecution. These findings contribute to previous literature that demonstrates how armed conflict, displacement, and violence exacerbate psychological distress among affected populations and more so among women. They also reveal the crucial role that resilience can play in preventing the burden of further psychological distress. Moreover, living situations (e.g., the different living conditions between Mam-Rashan and Shekhan) significantly impact psychological distress and resilience. These findings are critical and have multiple implications for humanitarian organizations, authorities, and responders who can consider the mentioned results when designing their responses and interventions. Additionally, the fact that subjective resilience has a significant negative association with distress in the camp with the worst reported mental health concerns should be more deeply analyzed in future studies.

### Limitations and future studies

Beyond the aforementioned limitation (e.g., cross sectional design), this study has additional limitations. While we successfully recruited an adequate sample size, we did not have a balanced number of participants concerning their demographics (e.g., marital status and educational background) to conduct reliable analyses to understand the contribution of each variable. In addition, we did not have preliminary data to compare with our results to observe the impact of the MHPSS interventions based on the type of MHPSS service provided.

Regarding future studies, we suggest that researchers include qualitative methods to collect data so that resilience contributors and coping strategies among participants can be more comprehensively explored. First, the qualitative style will reinforce understanding of resilience. This comprehension can be used to generalize skills and transfer them to other situations. Second, we recommend exploring MHPSS interventions and their contribution to building resilience among affected community members. Although we acknowledge the mentioned limitations, we are confident that the results from this study highlight the importance of subjective resilience in circumstances of psychological distress. They also establish the need to thoroughly analyze the meaning of perceived or subjective resilience and to consider sociodemographic variables when designing responses to humanitarian emergencies.

## Implications and conclusions

Armed conflict has a multi-layered impact on affected communities. It explicitly exacerbates psychological distress and daily stressors. Displacement, in particular, is considered a risk factor for mental health challenges. However, it is also vital to understand that not everyone in conflict contexts develops a psychological disorder. Contrarily, the majority of people bounce back and continue to live normally when provided access to adequate support. The findings from this study support the notion that resilience can act as a buffer in preventing further psychological distress. Therefore, it is critical that governmental and humanitarian agencies first consider the cultural lens of each community and reinforce related sources of strength for individuals and for the community support system. It is also crucial that the response to humanitarian crises does not undermine affected communities' sources of resilience. Thus, the typical western pathological interpretation of psychological disorders after a traumatic event might not adequately explain an individual's experiences in certain emergency contexts.

Furthermore, this study reveals that sex significantly impacts the status of psychological distress. Hence, it is critical to attend to differences in the needs of each individual and understand how gender should play a role in designing and providing services. Moreover, we established that living conditions (i.e., design of camps and available services) significantly influence psychological distress. Therefore, during emergencies, service providers and humanitarian agencies should enhance their understanding of each society and its dynamics, needs, and cultural context. A lesson that can be drawn from the current study is that scholars, humanitarian agencies, and related actors should further explore sources of strength among the Yazidis, build on the existing protective factors, and conduct work to enhance them.

We conclude that displacement significantly impacts psychological distress and also that certain groups and individuals (including women) are disproportionally affected based on sociodemographic characteristics. We also conclude that resilience plays a substantial role in predicting psychological distress. Thus, a comprehensive consideration of how each individual perceives their ability to overcome adversity could be useful for future research.

## Data Availability

The datasets generated and analyzed during the current study are available from the corresponding author on request.
